# Role of Zoo-Housed Animals in the Ecology of Ticks and Tick-Borne Pathogens—A Review

**DOI:** 10.3390/pathogens10020210

**Published:** 2021-02-16

**Authors:** Johana Hrnková, Irena Schneiderová, Marina Golovchenko, Libor Grubhoffer, Natalie Rudenko, Jiří Černý

**Affiliations:** 1 Centre for Infectious Animal Diseases and Zoonoses, Faculty of Tropical AgriSciences, Czech University of Life Sciences Prague, Kamýcká 129, Prague 6, 165 00 Suchdol, Czech Republic; jiricerny@ftz.czu.cz; 2 Department of Animal Science and Food Processing, Faculty of Tropical AgriSciences, Czech University of Life Sciences Prague, Kamýcká 129, Prague 6, 165 00 Suchdol, Czech Republic; schneiderova@ftz.czu.cz; 3 Department of Zoology, Faculty of Science, Charles University, Viničná 7, 2 128 00 Prague, Czech Republic; 4 Institute of Parasitology, Biology Centre, Czech Academy of Sciences, Branišovská 1160/31, 370 05 České Budějovice, Czech Republic; marina@paru.cas.cz (M.G.); liborex@paru.cas.cz (L.G.); natasha@paru.cas.cz (N.R.); 5 Faculty of Sciences, University of South Bohemia, Branišovská 1160/31, 370 05 České Budějovice, Czech Republic

**Keywords:** Ixodidae, ectoparasites, tick-borne diseases, tick hosts, zoo animals, exotic species, wildlife parks

## Abstract

Ticks are ubiquitous ectoparasites, feeding on representatives of all classes of terrestrial vertebrates and transmitting numerous pathogens of high human and veterinary medical importance. Exotic animals kept in zoological gardens, ranches, wildlife parks or farms may play an important role in the ecology of ticks and tick-borne pathogens (TBPs), as they may serve as hosts for local tick species. Moreover, they can develop diseases of varying severity after being infected by TBPs, and theoretically, can thus serve as reservoirs, thereby further propagating TBPs in local ecosystems. The definite role of these animals in the tick–host-pathogen network remains poorly investigated. This review provides a summary of the information currently available regarding ticks and TBPs in connection to captive local and exotic wildlife, with an emphasis on zoo-housed species.

## 1. Introduction

Ticks (Acari: Ixodidae) are arthropod ectoparasites, distributed worldwide. They are strictly hematophagous and feed on numerous terrestrial vertebrate species, including mammals, reptiles, birds and amphibians [1]. Studies suggest that, on a local scale, host selection of ticks and other ectoparasites is connected mainly with the ecological habitat they occupy [2,3,4]. Even though ticks are highly adaptable and able to colonize various habitats, they are usually recognized (mainly among the public) as parasites typically found in rural or forest areas. This notion is contradicted by several recent studies which showed that ticks are also frequently observed in urban and peri-urban habitats [5,6,7,8]. Typical urban areas inhabited by ticks include recreational areas, parks and cemeteries [9,10,11]. The increasing rate of urbanization worldwide facilitates the creation of ecotones which are ideal for the emergence of hotspots of tick-borne pathogens (TBPs) that might infect free-living, domesticated and possibly even zoo-housed animal species, potentially also endangering the urban human population [12,13,14]. Zoological gardens (zoos) are popular urban recreational areas with a semiforested or park-like character. The seminatural, fragmented environment characteristic for zoos is created to host various animal species with different habitat requirements. This is a factor that positively influences the life cycle of ticks and other ectoparasites [15,16,17,18]. That is why zoos are nowadays recognized as potential TBPs refugia [19,20,21,22]. Animal species kept in such refugia can therefore potentially serve as tick and TBPs reservoirs, allowing further propagation of TBPs within their local ecosystems. 

Indeed, several indigenous tick species have been reported in the areas of zoos, wildlife parks or farms worldwide. In the United States of America (USA) and Canada, *Ixodes pacificus* [23], *Ixodes scapularis* [24,25,26], *Amblyomma americanum*, *Rhipicephalus sanguineus* and *Dermacentor variabilis* [25,27,28] have been reported to exist in such captive exotic animal facilities. In Southern America, Brazilian zoo-animal infection cases have been connected to the following tick species of the *Amblyomma* and *Rhipicephalus* genera: *A. dubitatum*, *A. calcaratum*, *A. aureolatum*, *A. sculptum* or *R. sanguineus* in Southeastern regions of Brazil [21]. More *Amblyomma* species were collected from animals kept in zoos located in Northern and Northwestern Brazil: *A. dissimile*, *A. variatum*, *A. geayi*, *A. longirostre*, *A. goeldii*, *A. humerale*, *A. naponense* or *A. nodosum* [29,30]. In Europe, *Ixodes ricinus* is the most common tick found in zoos and wildlife parks or farms [20,31,32,33,34]. Nonindigenous tick species have been reported to feed on zoo animals, for example, the Asian tick, *Amblyomma javanense,* has been found on zoo-kept Asian water monitor (*Varanus salvator*) in South Carolina, USA [25].

All tick species belonging to the tick genera mentioned above (*Amblyomma, Dermacentor, Ixodes* and *Rhipicephalus*) develop in the three-host life cycle. The three-host life cycle is characteristic in its variability of host selection for each tick developmental stage (larvae, nymph and adult) [12]. Generally, the selection of natural hosts depends strongly on the development stage, in part due to different questing strategies connected to the position of the ticks on vegetation (how high each development stage can climb) [35]. Ideal hosts for tick larvae include small rodents like mice (for example *Peromyscus* spp. [36], *Apodemus* spp. [37]) or voles (for example *Myodes* spp. and *Microtus* spp. [37]), reptiles (like *Bothrops* spp. or *Dispas* spp. in Brazil [38]) and birds (for example migratory species like *Anthus trivialis* in Europe [39] or *Melospiza melodia* in the USA [40]). Such hosts are also suitable for nymphs. Both nymphs and larvae can also be found on larger animals like sheep, goats or other medium-sized animals [41,42]. Adult ticks frequently feed on larger animals, e.g., species of the Cervidae, Bovidae or Suidae families [35,42]. With each blood meal, ticks can acquire or spread various TBPs either by horizontal (stage-to-stage) transmission, vertical (female-to-egg) transmission or by cofeeding (nonsystemic) transmission [12,43,44]. Natural foci with the potential for emergence of TBPs represent a danger that is supported further by the ability of ticks and TBPs to adapt to host and habitat change [3,4,45,46]. 

## 2. Tick-Borne Pathogens in Zoo-Housed Animals

**Figure 1:**
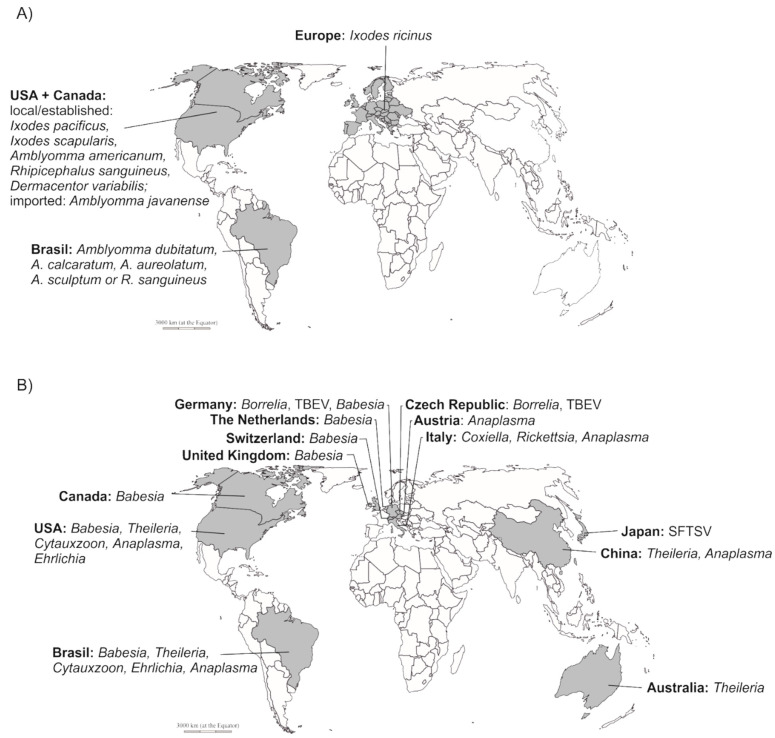
Ticks and tick-borne pathogens reported from zoo-housed animals: Ticks (**A**) or tick-borne pathogens (**B**) feeding on/detected in zoo-housed animals were found in all countries where this kind of research was performed. It indicates that zoo-housed animals may serve as hosts and reservoirs for local/established but also imported ticks and tick-borne pathogens. Nevertheless, lack of wider data and their anecdotal nature does not allow us to make definitive presumptions. Further research is needed to help us in understanding of the role of zoo-housed animals in tick biology. TBEV—tick-borne encephalitis virus. SFTSV—severe fever and thrombocytopenia syndrome phlebovirus

Infections caused by numerous TBPs have been reported in exotic (and local) animals under captive care in zoos, ranches, private farms and other similar facilities in many parts of the world (Figure 1). The various tick species that are able to transmit pathogens and are found in such facilities generally have well-studied vector capacity and competence for pathogens of medical and veterinary importance. Such key information can provide us with information regarding the risk of zoo-housed or urban-dwelling animals contracting tick-borne infections in a given geographic region. 

The tick species that belong to *I. ricinus* complex, which are predominant in Eurasian zoos and wildlife farms, i.e., *I. ricinus* and *Ixodes persulcatus*, are the primary vectors of Rickettsiales like *Anaplasma phagocytophilum* [47], tick-borne encephalitis virus (TBEV) [20,48,49], *Bartonella* spp., *Francisella tularensis*, multiple *Borrelia* spp. [20,49,50] and *Babesia* spp. [49,51]. The ticks commonly found in North American and Canadian zoos or ranches, i.e., *I. scapularis* and *I. pacificus*, are also recognized vectors of dangerous pathogens. Both *I. scapularis* and *I. pacificus* are known to transmit spirochetes from *Borrelia burgdorferi* sensu lato complex and *Borrelia myamotoi* [50], *Babesia microti*, *A. phagocytophilum*, *Ehrlichia muris*-like sp. or deer tick virus [52]. *A. americanum* and *D. variabilis* ticks are known vectors of *Cytauxzoon felis* [53]. *A. americanum* is also known vector of *Ehrlichia chaffeensis* [54], *Ehrlichia ewingii* [55], *Rickettsia amblyommii* and *Borrelia lonestari* [56]. *D. variabilis* transmits *Rickettsia rickettsii* - causative agent of Rocky Mountain spotted fever and other Rickettsiales [57]. *R. sanquineus*, found in Southern and Northern American zoos, were confirmed to transmit *Anaplasma platys*, *Hepatozoon canis*, *Cercopithifilaria* spp. [58,59,60], *Ehrlichia canis*, *Rickettsia massiliae*, *Rickettsia conorii* and *R. rickettsii* [59,60]. The majority of tick species found on animals housed in zoos and botanical gardens of Southern America, Brazil in particular, belong to the genus *Amblyomma*. In the Northern regions of Brazil *A. geayi, A. varium, A. longirostre* have been confirmed as vectors of *Rickttsia amblyommatis* [29,61,62,63,64]. *A. varium, A. nodosum and A. humerale* are able to transmit *Rickettsia bellii* [29,62,63,64,65]. *A. dissimile* was confirmed to carry *Rickettsia* sp. of the colombianensi strain [29,66], *A. nodosum* is also able to carry *Rickettsia parkeri*-like agent [29,65]. Further studies confirmed the presence of *A. sculptum* and *A. aureolatum* the main vectors of *R. rickettsii* (Brazilian spotted fever) in Southern regions of Brazil [21,67]. Other released results revealed the ability of *A. calcaratum* to vector the NOD strain of *Rickettsia* sp. [68] while *A. dubitatum* was confirmed to transmit several *Rickettsia* sp. [69] (see Figure 1 for an overview of the various tick species and their natural geographical distribution). These findings reveal the heightened risk for captive wildlife animals to be infected with the aforementioned pathogens. The risk of infection, however, is influenced by a large spectrum of factors including the reservoir capacity of the infected animal species or the presence of natural reservoir hosts of selected TBPs (for example, i.e. *Peromyscus leucopus*) that are able to thrive in urban environment [70]. The clinical manifestation of tick-borne diseases (TBDs) depends on the infected animal species; they can be hidden and nonspecific, which leads to underestimates of the epizootiology and pathology of many TBDs and their related issues among captive wildlife species. However, there are also reports of infections of tick-borne pathogens which have led to serious diseases and even fatalities, as will be discussed in this review.

## 3. Tick-Borne Encephalitis Virus (TBEV)

The TBEV can infect a wide range of mammals [71]. In humans, nonhuman primates, dogs and some rodent species, it can cause serious, and sometimes fatal, meningoencephalitis [72,73,74]. In ungulates, TBEV usually causes a subclinical infection, but the virus can be excreted into the milk of viremic individuals [75]. In rodents and insectivores, TBEV infection leads to long viremia without symptoms; this makes such species suitable reservoirs for the virus [71,76,77].

In 2006, a fatal case of TBEV infection was described in a female Barbary macaque (*Macaca sylvanus*) kept within the monkey enclosure of a zoo situated in southern Germany [78]. The monkey suffered staggering paresis of the hindlegs, incoordination and intermittent opisthotonos, before entering a coma four days after the onset of these symptoms. The comatose monkey was subsequently euthanized, and a post mortem necropsy, polymerase chain reaction (PCR) tests and histological tests confirmed an infection with TBEV. Even though this was the first described case of a natural TBEV infection in macaques, it was very similar to experimental infections of macaques used as model organisms for TBEV pathogenesis [78,79]. Later, serological tests were conducted on the remaining 283 macaques living within the same enclosure; among them, six (2.1%) were seropositive for anti-TBEV antibodies [72]. Anti-TBEV antibodies were also detected in sheep on the neighboring pastures, with a seroprevalence of 9% [72]. Similar cases could be prevented in the future, as macaques (and probably other primates) are likely to develop anti-TBEV immunity after vaccination with TBEV vaccines designed for human-use [80]. 

On the other hand, tests for anti-TBEV antibodies among other zoo animals were mostly negative, according to previous Czech zoological research results [20]. In this research, only two seropositive samples were recorded out of 133 tested serum samples from 69 animal species: one from a markhor (*Capra falconeri*) and one from a reindeer (*Rangifer tarandus*), as confirmed by both enzyme-linked immunosorbent assay and a neutralization test. 

## 4. Lyme Borreliosis Spirochetes

Lyme borreliosis (LB) spirochetes can cause systemic disease in humans, nonhuman primates, carnivores, ungulates and some rodent species [81,82,83], causing pathological changes in the skin, joints, heart and central nervous system [84,85]. However, clinical symptoms of LB in different animal species are variable [86,87]. They are influenced by, among other factors, the species of the *Borrelia* species and strain [88,89,90], as well as the host animal species and its breed. Different symptoms can be observed between horses [91,92], dogs [92,93,94] and natural hosts, like the white-footed mouse (*P. leucopus*) [90]. However, in many individuals, *Borrelia* infection symptoms are nonspecific, and asymptomatic infections are common in seropositive animals with lower antibody titers [87,91,94]. 

The prevalence of *Borrelia* among zoo animals has been investigated in Germany and the Czech Republic [19,20]. High numbers of *Borrelia*-infected individuals, or individuals having anti-*Borrelia* antibodies, were found in both studies. In the Czech Republic, DNA from spirochetes of the *B. burgdorferi* sensu lato complex was detected in a significant number of the tested vertebrate serum samples (69 positive cases, out of 133 tested samples – 51.8% affected). Those species with the highest number of positive samples were the Barbary sheep (*Ammotragus lervia*) with five positive samples (total sample size: n = 6), Grant’s zebra (*Eguus quagga boehmi*) also with five (n = 6), Hartmann’s mountain zebra (*Equus zebra hartmannae*) with four positive samples (n = 5), Grey wolves (*Canis lupus*) with four positives (n = 4) and Addax (*Addax nasomaculatus*) with five positive samples (n = 5) (Table 1; [20]). In Germany, sera from 1487 zoo animals were tested for the presence of anti-*Borrelia* antibodies. One hundred fifty-four samples (10.4%) were positive, while 168 samples (11.3%) produced borderline results. The highest number of positive samples was observed in Przewalski horses (*Equus przewalskii*), with 22 positives out of 98 tested animals, lions (*Panthera leo*), where 11 out of the 49 tested lions were positive, and forest buffalo (*Syncerus caffer nanus*), where four out of nine were positive ([19]; Table 1). Considering these studies [19,20,22], it is obvious that several animal species are susceptible to *Borrelia* infection. Among these are also the domestic goat (*Capra aegagrus f. hircus*), Barbary sheep (*A. lervia*), markhor (*C. falconeri*), mountain goat (*Oreamnos americanus*) and llama (*Lama guanicoe*) (Table 1). However, in some cases, the results of these studies varied. For example, in the German study, significant numbers of positive sera samples were found in domestic cattle (*Bos primigenius f. taurus*) and impala (*Aepyceros melampus)* [19]; however, in the Czech study, the sera of these animal species were negative [20]. On the other hand, the opposite was true for African wild dogs (*Lycaon pictus*) within the two zoos [19,20].

The serum complement of some animal species has a borreliacidal effect, which not only protects these animals from spirochete infection, but also purges *Borrelia* from infected ticks feeding on these animals [22,95,96]. This has a strong impact on the ecology of LB spirochetes within ecosystems where such animals are present. 

In research conducted by Ticha et al. [22], serum samples from zoo animals were tested for possible borreliacidal effects on three species of spirochetes from the *B. burgdorferi* sensu lato complex (*B. burgdorferi* sensu stricto (s.s.), *Borrelia garinii* and *Borrelia afzelii*). From the 135 tested serum samples from various zoo animals, 78 demonstrated some borreliacidal effect towards at least one of the tested *Borrelia* spp. The strongest borreliacidal effect was observed in the sera from the Burmese python (*Python bivittatus*), European rabbit (*Oryctolagus cuniculus*), radiated tortoise (*Astrochelys radiata*) and impala (*A. melampus*) (Table 1). Of all of the tested sera, only some showed borreliacidal effects toward all three tested *Borrelia* spp., as showed in Table 1. Most samples possessed selected resistance (resistance only towards one or two of the tested *Borrelia* types) or were sensitive to the studied *Borrelia* species. Sera from most of the carnivores, even-toed ungulates, rodents and some reptiles, showed only weak borreliacidal effects on the tested spirochetes.

The absence of a borreliacidal effect in the sera of some zoo animals could be an indication of their permissiveness to *Borrelia* infections, suggesting that these animals can theoretically serve as *Borrelia* reservoirs. On the other hand, animals whose sera have strong borreliacidal effects should be resistant to *Borrelia* infection. Unfortunately, no tests were conducted to assess whether these animals could also resolve *Borrelia* in the infected ticks feeding on them. 

## 5. Babesia, Theileria and Cytauxzoon Piroplasmida 

*Babesia* species are often observed in captive or semicaptive cervids and bovids [24,34,97,98,99], but they have also been found in other captive animal species. These protozoan parasites can complete their life cycle within multiple tick species, including *I. scapularis* [98,99], *I. ricinus* [97,100], *Dermacentor albipictus* [101], *A. americanum* [24] and *I. pacificus* [99]. Babesiosis has a range of typical symptoms, like hemolytic anemia, jaundice, fever, shaking and hemoglobinuria [102]. However, an asymptomatic disease course is also possible, especially in animals with a well-developed immunity [98]. 

Cases of acute babesiosis in nonindigenous cervids were reported in 2009 and 2012 in Germany [34]. In response, a nation-wide project was conducted in 2013, where samples were collected from 16 zoos located across the country [34]. This survey resulted in the detection of *Babesia capreoli, Babesia divergens, Babesia venatorum, Theileria* spp. and one unidentifiable *Babesia* sp., in captive reindeer (*R. tarandus*). Of the 123 tested reindeer samples, 29 were positive (23.6%), and 12 of the 16 facilities harbored at least one reindeer that tested positive for *Babesia* spp. [34]. 

Other babesiosis outbreaks were recorded in the Netherlands in 2011 and 2015 [97,103]. In 2011, a captive-bred forest reindeer calf died due to an acute *B. venatorum* infection [103]. In 2015, five out of 14 reindeer kept in the Ouwehand Zoo tested positive for *B. capreoli*, either through PCR testing or blood smears. Of the five positive animals, two died, one was euthanized and another animal died without testing positive for *Babesia* spp. All the mortalities were among young calves, born in the year of the outbreak, or in 2014. The surviving individuals with positive test results were adult females with no clinical signs of disease [97]. In Switzerland, a case report was published in 2019 identifying a young, captive reindeer calf with severe babesiosis infection clinical signs as positive for *B. venatorum* [32]. In Great Britain, seven fatal cases of babesiosis were confirmed in captive reindeer between the years 1997 and 1998, and *B. divergens* was identified as the probable causative species [104]. 

Canada and USA have also reported several acute and subclinical cases of babesiosis. Two fatal cases were reported in Canada in 2012, involving *Babesia* spp. isolated from captive-bred adult wapiti (*Cervus canadensis*) [105]. Between the years 2013 and 2016, nine fatal babesiosis cases were detected in Canadian zoo-kept adult reindeer and wapiti [24]. A higher number of positive cases were observed in Canada between the years 2016 to 2018 in zoo, or farm-kept cervids, like wapiti and red deer (*Cervus elaphus*) [98]. In the USA, fatal babesiosis infections were described very early in captive-bred cervids, including in caribou (*Rangifer tarandus caribou*) at the Minnesota Zoo [106] and the North American elk (*Cervus elaphus canadiensis*) kept on a farm in Texas [101]. Other severe American cases of babesiosis were observed in 2003 (semicaptive, adult North American elk; [107]) and in 2005 (adult captive reindeer from New York zoo; [108]). The study from New York zoo also identified three asymptomatic hosts of *B. odocoilei*: Yak (*Bos grunniens*), muntjac (*Muntiacus reevesi*) and markhor (*C. falconeri*) [108]. 

All the aforementioned Canadian and American studies related the positive samples to *Babesia odocoilei.* This *Babesia* species seems to be predominant in Canadian and North American captive cervids [24,98,105,107]. Considering these cases, it can be reasonably assumed that babesiosis is yet another global, tick-borne related threat to captive cervids. 

However, babesiosis infection is not exclusive to cervids and bovids; research conducted in several Brazilian zoos showed the prevalence of babesiosis in zoo felids, canids and a genet (*Genetta tigrina*). Most animals were seropositive for *Babesia canis*, but some (*Oncifelis colocolo* and the genet) were positive for a *Babesia* sp. with close similarity to *Babesia leo*, according to DNA testing [109]. In addition, free-roaming domestic cats in Brazil often stray into zoo areas and are therefore considered potential carriers of babesiosis. Both *Babesia* sp. (*Babesia vogeli*) and *Theileria* sp. were confirmed in some of the tested cat samples in the same area as these Brazilian zoo animals [110]. In Kansas, USA, an unknown *Babesia* spp. was observed in maned wolves (*Chrysocyon brachyurus*) in 2012 (first occurrence) and again in 2019. Both cases had severe clinical symptoms, and one case (2012) was fatal, even after treatment [111,112].

Piroplasms of the genera *Theileria* and *Cytauxzoon* are also dangerous protozoan parasites. *Theileria* have been observed in many tick species, including *Amblyomma* spp., *Haemahysalis* spp., *Rhipicephalus* spp. and *Hyalomma* spp. [113], whereas *Cytauxzoon* has been found in *Dermacentor* spp. [113,114,115]. *Theileria* species are variable in their pathogenesis and lifecycles; there are the so-called “transforming” species (*T. annulata, T. parva, T. lestoquardi, T. taurotragi* etc.) and the “nontransforming” species (*T. orientalis, T. mutans, T. cervi* and *T. velifera*) [113,116,117]. The transforming *Theileria* species have the ability to influence host leucocytes, causing them to enable unlimited proliferation of infected cells [116], resulting in symptoms such as polyphagia followed by anorexia, nasal discharge, fever, anemia, febrile generalized lymphadenopathy and hemorrhaging on the mucous membranes of the buccal cavity and conjunctiva [113,118]. Such an infection may be fatal. The nontransforming species lack the ability to cause proliferation, resulting mostly in benign infections with mild symptoms [116]. These symptoms can become chronic, causing anemia or persistent subclinical infections [119]. *Theileria* infections vary in terms of symptoms, depending on the infected animal species and the *Theileria* species. Besides from the free-roaming cats of Brazil [110], an unknown *Theileria* spp. was detected in Missouri, USA, in an adult male captive reindeer (*R. tarandus* [120]). Infections of South American tapirs (*Tapirus terrestris*) with *Theileria equi* were confirmed in zoo and botanical gardens located in Northern parts of Brazil [121]. *Theileria bicornis* was detected in samples of captive white rhinoceros (*Ceratotherium simum*) and black rhinoceros (*Diceros bicornis*) in Australian zoo [117]. A *Theileria* spp. was also found in the blood sample of one captive reindeer (*R. tarandus*) kept in a German zoo [34].

*Cytauxzoon felis* is a parasite of felids, of both wild and domestic origin. This parasite has been observed on several occasions in samples from zoo felids reared in Brazil, i.e., in ocelots (*Leopardus pardalis*) [114,122], lions (*P. leo*) [115], pumas (*Puma concolor*) and jaguars (*Panthera onca*) [114]. In Florida, USA, a white tiger (*Panthera tigris*) housed in a private breeding facility was also reported as positive for *C. felis* [123]. Cytauxzoonosis infection can be asymptomatic [114], but also fatal [115,123]. The disease has two phases: erythrocytic and macrophagic [124]. The erythrocytic phase is usually connected to anemia, while the macrophagic phase is marked by systemic circulatory obstructions, caused by schizont macrophages, and presents clinical signs such as anorexia, depression, dehydration, fever, icterus and dyspnea [124,125].

## 6. Rickettsiales

The bacteria of the order Rickettsiales cause a variety of diseases of veterinary and medical importance, including bovine anaplasmosis, human ehrlichiosis, Rocky Mountain spotted fever and scrub typhus [126]. Within the order Rickettsiales, the genera *Rickettsia*, *Ehrlichia and Anaplasma* are dependent on tick vectors like *A. americanum*, *R. sanguineus*, *D. variabilis, Ixodes* spp., *Haemaphysalis* spp., *Hyalomma* spp. and *Aponomma* spp. [23,26,28,31,127,128]. Various, and often nonspecific, clinical symptoms are associated with Rickettsiales infections in animals (anorexia, depression, dehydration, fever, lethargy, lymphadenopathy and ataxia) [23,26,28,129]. Acute infections with bacteria from the Anaplasmataceae family (*Anaplasma*, *Ehrlichia*) can be detected using blood smears, showing a characteristic “morulae” (mulberry-shaped microcolonies) located in the host cell cytoplasm [26,28,128].

In Europe, several reports have confirmed positive cases for Rickettsiales. *A. phagocytophilum* has been found in blood samples of captive reindeer (*R. tarandus*) kept in German zoos [33]. Furthermore, an asymptomatic lion (*P. leo*) was positive for an infection with *Rickettsia* sp. and *A. phagocytophilum* in Italy [130]. Acute anaplasmosis (*A. phagocytophilum*) was observed in captive timber wolves (*Canis lupus occidentalis*) in Austria [31].

In the USA, several cases were also reported for anaplasmosis (*A. phagocytophilum*), in four captive Przewalski’s horses (*E. przewalskii*) from Virginia [26]. *E. chaffeensis* was found in five ring-tailed lemurs (*Lemur catta*) and one ruffed lemur (*Varecia variegate rubra*) in the Duke Lemur Center in North Carolina (USA; [28]). *A. phagocytophilum* (under the old nomenclature of *Ehrlichia equi* in the case report) was confirmed in llama (*Lama glama*) from California, USA [23] and lastly, canine ehrlichiosis was noted in Florida, USA, in wolves, dogs and wolf-dog crosses [27].

Substantial research from Brazilian zoos showed that *Ehrlichia canis* was found in the following captive felids: jaguars (*P. onca*), ocelots (*L. pardalis*), jaguarundi (*Puma yagouaroundi*) and little spotted cats (*Leopardus tigrinus*). In this research, antibodies were found in four felids: two jaguarundi, one little spotted cat and one margay (*Leopardus wiedii*; [131]). Another study from Brazil confirmed that antibodies for *E. canis* existed in captive ocelots [122]. Further studies from André et al. [132] confirmed *Ehrlichia* spp. in captive canids, including European wolves (*C. lupus*), bush dogs (*Speothos venaticus*) and crab-eating foxes (*Cerdocyon thous*). Pumas (*P. concolor*), little spotted cats (*L. tigrinus*), ocelots (*L. pardalis*), jaguarundis (*P. yagouaroundi*), tigers (*P. tigris*) and lions (*P. leo*) also tested positive for *Ehrlichia* spp. Furthermore, *Anaplasma* spp. was confirmed in bush dogs and little spotted cats [132]. Three free-roaming cats surrounding the Brazilian zoo also tested positive for *Anaplasma* spp., which is closely related to *A. phagocytophilum* [110], showing that local animals can be a source of tick-borne pathogens that are then transferred to zoo-kept animals.

## 7. Coinfections with Multiple and Less Common Pathogens

In a report of Zhang et al. [133], novel *Theileria* spp., together with *A. phagocytophilum* and *Anaplasma bovis*, were found in the post mortem dissection of a one-year old South African giraffe (*Giraffa camelopardalis giraffa*), which was kept in Zhengzhou Zoo, China. The animal died suddenly, one day after the onset of severe clinical symptoms [133]. Another coinfection was observed in a lion (*P. leo*) in the Fasano Safari park in Italy. The animal tested positive for *Coxiella burnetii*, *Rickettsia* sp. and *A. phagocytophilum* [130]. In 2017, a rare emerging tick-borne virus causing severe fever and thrombocytopenia syndrome phlebovirus (SFTSV) was identified in two fatal cases in cheetah, infected in Hiroshima City Asa Zoological Park, Japan [134]. 

Regarding the aforementioned TBPs in zoo-housed and captive animals, Table 2 summarizes the prevalence, country of origin, animal species and collected tick species (excluding *Borrelia* spp. since these are discussed extensively in Table 1)

## 8. Conclusions and Recommendations

All of the aforementioned studies confirm the significant threat of ticks and tick-borne diseases to wild animals housed in zoos, wildlife parks or farms. Such zoo and zoo-like areas have been identified as being suitable for tick vectors and reservoir hosts of TBPs. The pathogens found in zoo-housed animals included viruses (TBEV, SFTSV), bacteria (*Borrelia*, *Anaplasma, Ehrlichia, Rickettsia* spp.) and protozoal parasites (*Babesia, Cytauxzoon* and *Theileria* spp.). It was confirmed that infection of the tick vectors with some of these pathogens, for example, *Borrelia* spp., TBEV, *Anaplasma* spp. and *Babesia* spp., increases the tick mobility, cold resistance, desiccation resistance and overall chance of survival [135]. There are other known tick-borne threats that are yet to be observed in zoo-housed animals, like the filariid nematode species *Cercopithifilaria* spp. and *Acanthocheilonema* spp. These parasites are frequently associated with dogs [136,137,138,139,140] and occasionally with wild-living animals [141]. They can be transmitted by various tick species, i.e., *Haemaphysalis flava*, *Haemaphysalis japonica* [141], *A. americanum* [142], *I. scapularis* [143,144] and *R. sanquineus* [145,146]. Focused sampling should be conducted to determine the potential spread of these parasites in zoos and other similar establishments. 

**Figure 2:**
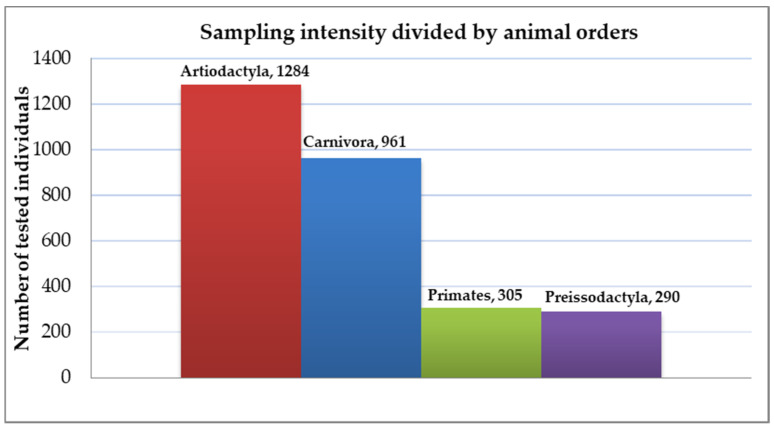
Differences in sampling for TBPs in zoo-housed animals (divided by orders). From this histogram, we can note the lack of testing in the Preissodactyla and Primates order. The orders Struthioniformes (2 samples), Phoenicopteriformes (1 sample), Testudines (1 sample), Squamata (1 sample) and Crocodilia (3 samples) have been tested only for *Borrelia* antibodies and in such small numbers that it would not provide any graphical value in the histogram. The role of these orders in the overall TBPs ecology is unclear; information is isolated only on the one tested pathogen. Some orders of animals, which are potentially threatened by TBPs and ticks, are omitted completely. For example: Chiroptera, Dermoptera, Edentata, Insectivora, Lagomorpha, Marsupialia, Proboscidea and Pholidata which all have the potential to carry ticks and therefore contract TBDs. Species from these orders are often kept in zoos in outdoor or semi-outdoor enclosures and the contact with ticks can occur. This suggests further need for the increase in research of the omitted or lacking animal orders.

Clinical manifestations of infections with the TBPs in captive animals can vary from unapparent to serious and even life threating [147]. It is clear that captive animals have variable sensitivities to the studied pathogens; however, it is not clear if zoo and farm-housed animals play a significant role as tick hosts and TBP reservoirs in their ecosystems. In the case of TBPs, most of them are probably incidental dead-end hosts, as they would not produce sufficient bacteremia/viremia for the infection of other ticks (although this question remains to be answered definitively). Figure 2 provides a summary of the amount of samples collected and tested across the several orders of zoo-housed animals (with connection to TBPs). More abundant sampling (Artiodactyla, Carnivora,) provides results that can be used to evaluate the role of these animal orders in the ecology of several TBPs. Data on Primates and Preissodactyla are insufficient to draw any wider conclusions in terms of overall TBP transmission, and they usually provide information about the incidence of only one pathogen (case reports). 

Some of the pathogens (TBEV, *Borrelia* spp., *A. phagocytophilum*, *E. cheffeensis*, *C. burnetii*) and tick species (*A. americanum*, *A. sculptum I. ricinus*, *I. scapularis*, *D. variabilis*) detected in zoos or zoo-like areas represent a notable threat to the health of humans that live nearby. Since zoos are places with high densities of humans, exotic animals, domestic animals and wildlife opportunists, they create ideal hotspots for the spread of TBPs, ticks and other ectoparasites [18]. The importance of surveillance and research of tick vectors and TBPs that exist in close proximity to human habitats is supported by the fact that the annual number of visitors to zoos is more than 700 million worldwide [148]. The already available evidence of tick-borne pathogens infecting zoo-housed animals should raise awareness of scientists, zookeepers, veterinarians, medical doctors and other specialists.

Another risk for zoo and other captive animals is free-roaming domestic cats that often stray into zoo or farm grounds. These cats are commonly infested with local ticks, and are hosts to various vector-borne infections [110,149,150,151]. They can thus potentially serve as one of the sources that increase the numbers of infected ticks in the areas that they commonly occupy. As a preventative measure, the activity of free-roaming domestic cats should be monitored and minimized in establishments where exotic animals are kept. Advanced preventative techniques in the forms of various vaccines are also available for the prevention of tick-borne infections in some animal species. In addition to the existing TBEV vaccine approved for human use, which was shown to be efficient for other primates [80], there is a borrelia vaccine approved for use in dogs [152]. Recently, this vaccine was tested on horses [153], and it could be expected that it may trigger protection in other animals too, at the very least, in canids. Furthermore, vaccines against bovid ticks from the genus *Rhipicephalus* were developed for use in cattle [154], and since the vaccine works in sheep as well, it can be expected that it may protect other ruminant species [154]. Also, landscape management with respect to tick-associated risks can help lower the prevalence of ticks, and subsequently, of TBPs, thus enhancing any other preventative measures taken [155].

In conclusion, ticks and TBPs present a challenge for a wide range of zoo, veterinary and public health experts. However, due to the poor understanding of the role of zoo animals in the biology of ticks and TBPs, further research in this area is clearly urgently required.

## 9. Other Potentially Tick-Borne Threats to Zoo-Housed and Captive Animals

Some pathogens are less specialized and spread through a wider range of vectors, e.g., vertebrates, mites, lice, mosquitoes and, of course, ticks. Even though some pathogens are less studied, they still represent a threat to both animal and human health.

Bacteria of the order Chlamydiales have been connected to Ixodid ticks for some time [156,157,158,159]. The most intensively studied is the Chlamydiaceae family. Other families are included in the order, but they are usually summarized under the term *Chlamydia*-like organisms (CLOs). These bacterial pathogens are causative agents of wide range of human and animal (some zoonotic) diseases [160]. Tick-borne CLO transmissions have been observed in humans [156], while various species of animals have been confirmed to harbor chlamydial agents, but without the direct connection to ticks. Among vertebrates, several species of bats (free-living and captive) have been found to be positive for a wide range of CLOs [161]. *Chlamydophila psittaci* has been found in the eyes of various livestock [162]. *Chlamydophila abortus* and *Chlamydophila pecorum* has been detected in a water buffalo (*Bubalus bubalis*) [163]. *Chlamydia felis* infection has been confirmed in cats and dogs [164], while *Chlamydiaceae* has been detected in domestic pigs (*Sus scrofa* f. *domestica*) [165]. These studies suggest the possibility of infection for both humans and captive/domestic animals living in their close vicinity.

Another potentially tick-borne pathogen that causes health problems is the bacteria *F. tularensis*. This pathogen can be transmitted through various sources: aerosol droplets, infected animal carcasses, contaminated food (alimentary transmission) or the bite of an infected arthropod [166,167]. *F. tularensis* can be transmitted by all tick life stages and horizontal transmission has been confirmed [167]. There have been positive cases of tularemia infection in animals in several zoological gardens. A fatal case in a Bornean orangutan (*Pongo pygmaeus*) was reported at Topeka Zoo, Kansas in 2003 [25], which was directly connected to tick bite. Several other zoos in North America have confirmed *F. tularensis* infections in other animal species: golden-lion tamarins (*Leontopithecus rosalia*), red-handed tamarin (*Saguinus midas)* [25], squirrel monkeys (genus *Saimiri*) [168], black and white-ruffed lemurs (*Varecia variegate)*, ring-tailed lemurs (*L. catta*), white handed gibbon (*Hylobates lar*) and greater spotnose guenon (*Cercopithecus nictitans*) [169]. *F. tularensis* infections have also been observed in animals in German zoos (in a wide range of animal species) [170]. Human and animal (tamarins and a talapoin monkey (*Miopithecus talapoin*)) cases have also been reported in Canada [171]. However, none of these studies provided any link to tick or other ectoparasite bites, so it remains unclear whether the connection exists. Nonetheless, it is still evident that zoo-housed animals and humans are threatened by this pathogen.

Bacteria of the genus *Bartonella* are known to cause various diseases, for example, the cat scratch disease in humans [172]. *Bartonella* spp. has been connected to several tick species [172,173,174,175]. Domestic cats are known reservoirs of *Bartonella* spp., e.g., *B. henselae*, *B. clarridgeiae* and *B. koehlerae* [176,177]. Samples from free-roaming domestic cats located in zoo areas in Brazil have been found to be positive for *Bartonella* spp. [110]. This could lead to spillover of this pathogen to the zoo tick population, even though the described infestation was most likely flea-borne [110]. Recently, tick-borne *Bartonella* spp. cases have been observed in dromedary camels (*Camelus dromedarius*) infected with *B. henselae* [178], domesticated yaks (*Bos grunniens*) [179] and in livestock animals like cattle [180,181], goats [181,182] and horses [182]. Some of these species, like dromedary camels or yaks, are often kept in zoos, so this information may be useful for the prevention of this potentially tick-borne disease. 

There are other widely known pathogens that are yet to be fully established as potentially tick-borne, e.g., the parasite *Toxoplasma gondii.* Even though this parasite is not usually associated with ticks, some studies have proved the ability of ticks to transmit it [183,184]. In conclusion, it should be noted even pathogens which are less commonly attributed to ticks and captive animals have the potential to cause serious damage.

## Figures and Tables

**Table 1:** The prevalence of *Borrelia* specific antibodies in exotic zoo animals in Czech Republic and Germany together with observed borrelicidal effect of zoo animal sera.

Group	Family	Animal Species	*Borrelia* Seroprevalence		Borrelicidal Effect		
			DE	CZ	*Borrelia burgdorferi s.s.*	*Borrelia garinii*	*Borrelia afzelii*
**Odd-toed ungulates**	Equidae	*Equus africanus f. asinus*	13/1 (8%)	2/2 (100%)	weak to moderate	moderate to strong	moderate to strong
		*Equus africanus somaliensis*	10/1 (10%)	1/1 (100%)	-*	-	-
		*Equus ferus caballus*	5/2 (40%)	3/3 (100%)	-	-	-
		*Equus ferus ferus*	-	5/5(100%)	-	-	-
		*Equus grevyi*	18/1 (6%)	-	-	-	-
		*Equus hemious kulan*	12/2 (17%)	-	-	-	-
		*Equus przewalskii*	98/22 (22%)	-	-	-	-
		*Equus quagga*	33/9 (27%)	8/7 (88%)	weak	strong	Strong
		*Equus zebra*	25/1 (4%)	5/4 (80%)	weak	strong	Strong
	Tapiridae	*Tapirus terrestris*	10/2 (20%)	-	-	-	-
	Rhinocerotidae	*Ceratotherium simum*	3/2 (67%)	-	-	-	-
		*Diceros bicornis*	-	7/0 (0%)	-	-	-
**Even-toed ungulates**	Cervidae	*Alces alces alces*	13/2 (15%)	1/1 (100%)	-	-	-
		*Cervus albirostris*	10/1 (10%)	-	-	-	-
		*Cervus canadensis*	-	1/1 (100%)	-	-	-
		*Cervus elaphus bactrianus*	11/0 (0%)	-	-	-	-
		*Cervus elaphus hippelaphus*	37/0 (0%)	-	-	-	-
		*Cervus eldi thamin*	10/1 (10%)	-	-	-	-
		*Cervus nippon pseudaxis*	20/0 (0%)	-	-	-	-
		*Cervus timorensis*	3/1 (33%)	-	-	-	-
		*Dama dama dama*	20/0 (0%)	-	-	-	-
		*Elaphurus davidianus*	14/0 (0%)	-	-	-	-
		*Moschus moschiferus*	4/3 (75%)	-	-	-	-
		*Rangifer tarandus*	13/1 (8%)	1/1 (100%)	-	-	-
	Camelidae	*Camelus ferus f. bactrianus*	14/1 (7%)	-	-	-	-
		*Lama guanicoe*	48/3 (6%)	1/1 (100%)	-	-	-
		*Lama vicugna*	5/1 (20%)	-	-	-	-
	Suidae	*Phacochoerus africanus*	-	1/0 (0%)	weak	weak	Weak
	Bovidae	*Addax nasomaculatus*	-	5/5 (100%)	-	-	-
		*Aepyceros melampus*	6/1 (17%)	3/0 (0%)	strong	strong	weak to strong
		*Ammelaphus imberbis*	-	2/2 (100%)	-	-	-
		*Ammotragus lervia*	19/1 (5%)	6/5 (83%)	moderate	weak	Moderate
		*Antidorcas marsupialis*	-	1/0 (0%)	-	-	-
		*Antilope cervicapra*	16/1 (6%)	-	-	-	-
		*Bison bison*	14/2 (14%)	-	-	-	-
		*Bison bonasus*	17/0 (0%)	-	-	-	-
		*Bos gaurus*	8/1 (13%)	-	-	-	-
		*Bos gaurus f. frontalis*	4/1 (25%)	-	-	-	-
		*Bos javanicus*	23/2 (9%)	-	-	-	-
		*Bos primigenius f. Taurus*	21/2 (10%)	2/0 (0%)	weak	strong	Moderate
		*Boselaphus tragocamelus*	7/2 (29%)	-	-	-	-
		*Bubalus arnee f. bubalis*	9/2 (22%)	-	-	-	-
		*Budorcas taxicolor*	11/3 (27%)	-	-	-	-
		*Capra aegagrus cretica*	9/1 (11%)	-	-	-	-
		*Capra aegagrus f. hircus*	17/4 (24%)	5/5 (100%)	-	-	-
		*Capra caucasica*	-	1/1 (100%)	-	-	-
		*Capra falconeri heptneri*	12/5 (42%)	1/1 (100%)	-	-	-
		*Capra ibex nubiana*	6/2 (33%)	-	-	-	-
		*Cephalophus natalensis*	1/1 (100%)	-	-	-	-
		*Connochaetes gnou*	-	3/1 (33%)	weak	strong	Weak
		*Damaliscus pygargus phillipsi*	-	1/1 (100%)	-	-	-
		*Eudorcas thomsonii*	-	2/2 (100%)	-	-	-
		*Gazella dama*	9/1 (11%)	-	-	-	-
		*Hemitragus jemlahicus*	10/0 (0%)	-	-	-	-
		*Hippotragus equinus*	-	1/1 (100%)	-	-	-
		*Hippotragus niger*	-	4/3 (75%)	moderate	strong	Strong
		*Kobus ellipsiprymnus*	11/1 (9%)	1/0 (0%)	moderate	moderate to strong	moderate to strong
		*Kobus leche*	-	1/1 (100%)	-	-	-
		*Naemorhedus caudatus*	-	2/0 (0%)	weak	moderate to strong	Moderate
		*Nanger dama*	-	5/3 (60%)	weak to moderate	moderate	Moderate
		*Oreamnos americanus*	20/9 (45%)	2/2 (100%)	-	-	-
		*Oryx gazella dammah*	10/0 (0%)	5/3 (60%)	moderate	strong	weak to strong
		*Oryx gazella gazella*	10/0 (0%)	2/2 (100%)	-	-	-
		*Ovibos moschatus*	11/8 (72%)	-	-	-	-
		*Ovis ammon f. aries*	83/8 (10%)	5/3 (60%)	moderate	strong	Moderate
		*Ovis ammon musimon*	18/3 (17%)	-	-	-	-
		*Ovis dalli*	3/1 (33%)	-	-	-	-
		*Ovis nivicola*	1/1 (100%)	-	-	-	-
		*Pseudois nayaur*	11/0 (0%)	-	-	-	-
		*Redunca redunca*	14/0 (0%)	1/0 (0%)	strong	strong	Weak
		*Saiga tatarica*	31/1 (3%)	-	-	-	-
		*Syncerus caffer caffer*	17/2 (12%)	1/0 (0%)	weak	weak	Weak
		*Syncerus caffer nanus*	9/4 (44%)	-	-	-	-
		*Tragelaphus angasii*	-	2/1 (50%)	weak	weak	Weak
		*Tragelaphus strepsiceros*	10/0 (0%)	2/2 (100%)	-	-	-
	Giraffidae	*Giraffa c. reticulate*	-	1/0 (0%)	moderate	strong	Strong
		*Giraffa c. rothschildi*	-	2/0 (0%)	moderate	strong	Strong
**Carnivores**	Felidae	*Acinonyx jubatus*	-	1/0 (0%)	weak	weak	Weak
		*Crocuta crocuta*	-	1/1 (100%)	-	-	-
		*Felis lybica*	4/1 (25%)	-	-	-	-
		*Felis serval*	3/1(33%)	1/0 (0%)	weak	weak	Weak
		*Lynx rufus*	2/1 (50%)	-	-	-	-
		*Panthera leo*	49/11 (22%)	1/0 (0%)	weak	weak	Weak
		*Panthera leo persica*	-	1/0 (0%)	weak	moderate	Weak
		*Panthera onca*	15/1 (7%)	-	-	-	-
		*Panthera pardus*	59/8 (14%)	-	-	-	-
		*Panthera pardus orientalis*	-	1/0 (0%)	weak	weak	Weak
		*Panthera tigris*	98/2 (2%)	-	-	-	-
		*Puma concolor*	12/0 (0%)	-	-	-	-
	Ursidae	*Ursus arctos arctos*	11/0 (0%)	-	-	-	-
		*Ursus maritimus*	12/0 (0%)	-	-	-	-
		*Ursus thibetanus*	6/1 (17%)	-	-	-	-
	Canidae	*Canis lupus*	-	4/4 (100%)	-	-	-
		*Canis mesomelas*	-	1/1 (100%)	-	-	-
		*Lycaon pictus*	14/0 (0%)	2/1 (50%)	weak	weak	Weak
	Otariidae	*Zalophus californianus*	1/1 (100%)	-	-	-	-
**Primates**	Cercopithecidae	*Colobus angolensis*	-	1/0 (0%)	-	-	-
	Hylobatidae	*Hylobates lar*	-	1/1 (100%)	-	-	-
**Birds**	Phoenicopteridae	*Phoenicopterus roseus*	-	1/1 (100%)	weak	weak	Strong
	Struthionidae	*Struthio camelus*	-	2/0 (0%)	weak	weak	Strong
**Reptiles**	Testudinidae	*Astrochelys radiata*	-	1/0 (0%)	strong	strong	Strong
	Crocodylidae	*Crocodylus siamensis*	-	3/0 (0%)	weak	weak	Weak
	Pythonidae	*Python bivittatus*	-	1/0 (0%)	strong	strong	Strong

DE—Germany, CZ—Czech Republic, * hyphens in the table represent unavailable data in given research.

**Table 2:** Ticks and tick-borne diseases detected in animals living in zoos and zoo-like establishments.

Pathogen	Animal Species	Tick Species Found	Prevalence (Positive/Tested)	Country	Reference
TBEV	Barbary macaque (*Macaca sylvanus*)	*Ixodes ricinus*	8/284 (2.8%)	Germany	[72,78]
	Markhor (*Capra falconeri)*	*I. ricinus*	1/1 ab* (100%)	Czech Republic	[20]
	Reindeer (*Rangifer tarandus*)	*I. ricinus*	1/1 ab (100%)	Czech Republic	[20]
*Babesia* spp.	Ocelot (*Leopardus pardalis*)	N/A	26/43 ab (60.5%)	Brazil	[109]
	Little-spotted cat (*Leopardus tigrinus*)	N/A	9/38 ab (23.7%)	Brazil	[109]
	Margay (*Leopardus wiedii*)	N/A	2/4 ab (50%)	Brazil	[109]
	Pampas cat (*Oncifelis colocolo*)	N/A	3/5 ab (60%)	Brazil	[109]
	Jaguar (*Panthera onca*)	N/A	6/13 ab (46.1%)	Brazil	[109]
	Puma (*Puma concolor*)	N/A	2/18 ab (11.1%)	Brazil	[109]
	Jaguarundi (*Puma yagouaroundi*)	N/A	6/25 ab (24%)	Brazil	[109]
	Crab-eating fox (*Cerdocyon thous*)	N/A	2/39 ab (5.1%)	Brazil	[109]
	Bush dog (*Speothos venaticus*)	N/A	8/27 ab (29.6%)	Brazil	[109]
	Maned wolf (*Chrysocyon brachyurus*)	N/A	2/2 (100%)	USA	[111,112]
	Reindeer (*R. tarandus*)	N/A	1/1 (100%)	USA	[106]
*Babesia odocoilei*	Wapiti (*Cervus canadensis*)	N/A	2/30 (6.7%)	Canada	[98,105]
	Reindeer (*R. tarandus*)	speculated *Ixodes scapularis*	12/12 (100%)	Canada, USA	[24,108]
	Red deer (*Cervus elaphus*)	N/A	4/144 (2.8%)	Canada, USA	[98,101,107]
	Markhor (*C. falconeri*)	speculated *I. scapularis*	4/6 (66.7%)	USA	[108]
	Yak (*Bos grunniens*)	speculated *I. scapularis*	1/2 (50%)	USA	[108]
	Muntjac (*Muntiacus reevesi*)	speculated *I. scapularis*	1/2 (50%)	USA	[108]
*Babesia venatorum*	Reindeer (*R. tarandus*)	*I. ricinus*	21/141 (14.9%)	Germany, Netherlands, Switzerland	[32,34,103]
*Babesia capreoli*	Reindeer (*R. tarandus*)	*I. ricinus*	7/137 (5.1%)	Germany, Netherlands	[34,97]
*Babesia divergens*	Reindeer (*R. tarandus*)	*I. ricinus*	7/154 (4.5%)	Germany, Great Britain	[34,104]
*Babesia capreoli*-like	Reindeer (*R. tarandus*)	*I. ricinus*	4/123 (3.3%)	Germany	[34]
*Babesia odocoilei*-like	Reindeer (*R. tarandus*)	*I. ricinus*	2/123 (1.6%)	Germany	[34]
*Babesia leo*	Genet (*Genetta tigrina*)	N/A	1/2 (50%)	Brazil	[109]
*Theileria* spp.	Reindeer (*R. tarandus*)	N/A	1/1 (100%)	USA	[120]
	Reindeer (*R. tarandus*)	*I. ricinus*	1/123 (0.8%)	Germany	[34]
*Theileria equi*	Tapir (*Tapirus terrestris*)	N/A	11/19 (57.9%)	Brazil	[121]
*Theileria bicornis*	White rhinoceros (*Ceratotherium simum*)	N/A	2/2 (100%)	Australia	[117]
	Black rhinoceros (*Diceros bicornis*)	N/A	1/7 (14.3%)	Australia	[117]
*Cytauxzoon felis*	Ocelot (*L. pardalis*)	N/A	7/138 (5%)	Brazil	[114,122],
	Puma (*P. concolor*)	N/A	2/9 (22.2%)	Brazil	[114]
	Jaguar (*Panthera onca*)	N/A	1/9 (11.1%)	Brazil	[114]
	Lion (*Panthera leo*)	*Amblyomma cajennense*	1/1 (100%)	Brazil	[115]
	Tiger (*Panthera tigris*)	*Amblyomma americanum*	1/1 (100%)	USA	[123]
*Anaplasma phagocytophilum*	Reindeer (*R. tarandus*)	*I. ricinus*	17/123 (13.8%)	Germany	[33]
	Przewalski’s horse (*Equus przewalskii*)	unspecified Ixodid ticks	4/4 (100%)	USA	[26]
	Lion (*P. leo*)	N/A	1/10 (10%)	Italy	[130]
	Timber wolf (*Canis lupus occidentalis*)	*I. ricinus*	1/1 (100%)	Austria	[31]
	Llama (*Lama glama*)	*Ixodes pacificus*	1/1 (100%)	USA	[23]
	Little-spotted cat (*L. tigrinus*)	N/A	4/25 (16%)	Brazil	[132]
	Bush dog (*Speothos venaticus*)	N/A	1/27 (3.7%)	Brazil	[132]
*Ehrlichia canis*	Jaguar (*P. onca*)	N/A	2/9 (2.2%)	Brazil	[131]
	Ocelot (*L. pardalis*)	N/A	3/30 (10%)	Brazil	[122,132]
	Jaguarundi (*P. yagouaroundi*)	N/A	5/25 ab (20%)	Brazil	[131,132]
	Little-spotted cat (*L. tigrinus*)	N/A	5/39 ab (12.8%)	Brazil	[131,132]
	Margay (*Leopardus wiedii*)	N/A	1/1 ab (100%)	Brazil	[131]
	Puma (*P. concolor*)	N/A	3/17 (17.6%)	Brazil	[131,132]
	Pampas cat (*L. colocolo*)	N/A	1/3 (33.3%)	Brazil	[131]
	Lion (*P. leo*)	N/A	2/12 (16.7%)	Brazil	[132]
	Crab-eating fox (*C. thous*)	N/A	3/39 (7.7%)	Brazil	[132]
	Bush dog (*S. venaticus*)	N/A	5/27 (18.5%)	Brazil	[132]
	Timber wolf (*Canis lupus*)	*Rhipicephalus sanquineus*	13/17 (76.5%)	USA	[27]
*Ehrlichia chaffeensis*	Ring-tailed lemur (*Lemur catta*)	*A. americanum*	7/9 (77.8%)	USA	[28]
	Ruffed lemur (*Varecia variegate rubra*)	*A. americanum*	1/10 (10%)	USA	[28]
	Little-spotted cat (*L. tigrinus*)	N/A	3/25 (12%)	Brazil	[132]
	Ocelot (*L. pardalis*)	N/A	2/15 (13.3%)	Brazil	[132]
	Puma (*P. concolor*)	N/A	2/8 (25%)	Brazil	[132]
	Tiger (*P. tigris*)	N/A	2/8 (25%)	Brazil	[132]
	Jaguarundi (*P. yagouaroundi*)	N/A	1/19 (5.3%)	Brazil	[132]
	Lion (*P. leo*)	N/A	1/12 (8.3%)	Brazil	[132]
	European wolf (*C. lupus*)	N/A	1/3 (33.3%)	Brazil	[132]
	Crab-eating fox (*C. thous*)	N/A	2/39 (5.1%)	Brazil	[132]
*Rickettsia* spp.	Lion (*P. leo*)	N/A	2/10 (20%)	Italy	[130]
*Theileria* spp., *A. phagocytophilum* and *A. bovis*	South African giraffe (*Giraffa camelopardalis giraffa*)	N/A	1/1 (100%)	China	[133]
*Coxiella burnetii* and *A. phagocytophilum*	Lion (*P. leo*)	N/A	1/1 (100%)	Italy	[130]
SFTSV	Cheetah (*Acinonyx jubatus*)	unspecified Ixodid tick	2/2 (100%)	Japan	[134]

specific data. ab*: antibodies positive; without ab: PCR positive; N/A: No ticks found on the positive animals.

## Data Availability

No new data were created or analyzed in this study. Data sharing is not applicable to this article.
